# Gluten Sensitivity Presenting as a Neuropsychiatric Disorder

**DOI:** 10.1155/2014/293206

**Published:** 2014-02-12

**Authors:** Stephen J. Genuis, Rebecca A. Lobo

**Affiliations:** ^1^Faculty of Medicine, University of Alberta, Edmonton, AB, Canada T6K 4C1; ^2^Department of Family Medicine, University of Alberta, Edmonton, AB, Canada T6G 2C8

## Abstract

There has been increasing recognition in the medical community and the general public of the widespread prevalence of gluten sensitivity. Celiac disease (CD) was initially believed to be the sole source of this phenomenon. Signs and symptoms indicative of nonceliac gluten sensitivity (NCGS), in which classical serum and intestinal findings of CD may be absent, have been frequently reported of late. Clinical manifestations in patients with NCGS are characteristically triggered by gluten and are ameliorated or resolved within days to weeks of commencing a gluten-free diet. Emerging scientific literature contains several reports linking gluten sensitivity states with neuropsychiatric manifestations including autism, schizophrenia, and ataxia. A clinical review of gluten sensitivity is presented alongside a case illustrating the life-changing difference achieved by gluten elimination in a patient with a longstanding history of auditory and visual hallucinations. Physicians in clinical practice should routinely consider sensitivity issues as an etiological determinant of otherwise inexplicable symptoms. Pathophysiologic mechanisms to explain the multisystem symptomatology with gluten sensitivity are considered.

## 1. Introduction

Over the last decade, there have been increasing reports of myriad adverse reactions associated with gluten exposure. Classical manifestations of gluten intolerance are those of celiac disease (CD), including gastrointestinal upset, failure to thrive, weight loss, and anemia. However, emerging scientific literature has noted a link between gluten ingestion and symptomatology from nearly every organ system, often in the absence of classic histological findings of CD on intestinal biopsy.

It has been hypothesized for quite some time that gluten sensitivity may also impair central nervous system functioning [1]. In 1996, Hadjivassiliou et al. found a significant difference in the prevalence of patients with positive antigliadin antibodies amongst those with neurological symptoms of unknown cause (57%) compared to a control group of healthy patients (12%) [1]. Amid the 57% who did have positive antibody titres, the majority did not demonstrate histological evidence diagnostic of celiac disease. In a 2010 article published in *Lancet Neurology*, Hadjivassiliou and colleagues published additional support for the link between gluten sensitivity and neurological manifestations, including ataxia, neuropathy, encephalopathy, epilepsy, myopathy, and myelopathy [2]. Similar results continue to be reported in the medical literature and give credence to the association between gluten sensitivity and neurological symptoms in the absence of celiac disease [3–6].

In this paper, a clinical review of gluten sensitivity as it relates to mental health is presented for consideration as well as proposed pathophysiological mechanisms for the multimorbidity and neuropsychiatric symptoms associated with gluten sensitivity in some patients. A case history of a 23-year-old female with a longstanding history of auditory and visual hallucinations is initially presented as an example of what can be clinically achieved in gluten sensitive individuals following elimination of the inciting food trigger.

## 2. Case Report

From birth, the patient was described by her mother as a colicky but otherwise healthy infant. Around age 4 or 5, however, the child began to experience recurrent gastrointestinal problems, as well as the onset of frequent visual and auditory hallucinations. She recalls that she would often “see beings and, at times, entire scenes, that no one else would see.”

The nature of the hallucinations varied considerably. For example, her hallucinations ranged from explicit scenes of an 8-year-old, blonde haired boy named Tommy, who the patient believed to be a child her mother had miscarried, to ghosts and colorful fairies that communicated with her and slept in trees outside her window. These happy scenarios were sharply contrasted at times by terrifying hallucinations of hideous creatures covered in burns and boils who would threaten endless torture. Her hallucinations would sometimes be colored with religious themes and would solicit action on her part. For example, she recalls seeing beautiful, elaborate banquets in heaven with sparkling crystal goblets and hearing what she described as God's voice asking her to do specific, though seemingly random, tasks such as planting 3 plants in a particular part of the garden.

Some of her hallucinations were recurrent and many involved interaction. These experiences occurred on a nearly daily basis and were “just a part of (her) life.” Other than the visions of terror, she quite enjoyed her discussions with the majority of these unknown figures. The patient relates that these hallucinations were indistinguishable from reality, and at times she would physically reach out to touch the different characters without realizing they were not real.

The patient describes relying fairly heavily on these imaginary characters for companionship as a child and claims that they inhibited her ability to form friendships with other children. As she grew older, she also found these hallucinations quite distracting rendering her unable to concentrate adequately at school or to study for exams. After the patient disclosed these hallucinations to her mother, religious counsel was initially sought where it was suggested that the hallucinations were perhaps attributable to divine causes. Although the patient came from a loving and supportive family, these symptoms were never brought to medical attention.

The hallucinations as well as the gastrointestinal symptoms continued through her childhood and teen years, causing her to miss considerable amounts of school. After disclosing her abdominal symptoms, she was diagnosed by a physician with irritable bowel syndrome and was started on a daily regimen of high dose psyllium. On her own initiative, she began to experiment with elimination diets. She progressively eliminated soy, corn, and dairy but reported no change in any of her symptoms.

She began university and states it was a “miracle (she) never failed out of school” and maintained a C average. While at university, she met a young man and entered a romantic relationship with him. Although quite smitten, she reports that relapsing hallucinations took a toll on their relationship as she was, at times, affected to the point of not recognizing her boyfriend.

### 2.1. Dietary Manipulation

After attending nutrition lectures with her partner, the patient was introduced to the idea of gluten sensitivity and decided to abstain from gluten exposure. After eliminating gluten in September 2009, her gastrointestinal symptoms and hallucinations completely abated, and she felt an improvement in her ability to concentrate at school. She describes being able to sit down with sustained focus on study for the first time in her life, leading to the completion of her biology degree and the obtainment of employment.

Given the dramatic resolution of her symptoms, the patient chose to subsequently continue to remain entirely gluten-free. Despite her efforts, however, she occasionally experienced inadvertent gluten exposures, which triggered a clear reproduction of her previous symptoms including vivid hallucinations and severe abdominal pain. Exposure to gluten in each case involved traces contained in contaminated food rather than a willful transgression with copious ingestion of gluten. During these episodes she was sometimes with her partner, who noted she became completely disoriented and did not recognize familiar surroundings. For example, 3 hours after unintentionally eating gluten-containing oatmeal, she began to see “aliens” in the computer screens at work and believed they had restrained a teddy bear in some of the computer cords. She unplugged many of the apparatuses in the office in an attempt to rescue the stuffed animal. When her partner arrived to pick her up from work, she did not recognize him at all and was very confused about how he had a key to her apartment. The symptoms began to abate after 24 hours and completely resolved within 2-3 days.

The patient reports that this pattern was predictable. When reexposed to gluten, relapse consistently occurred within 3–5 hours and would result in significant disorientation and departure from reality. The episodes spontaneously resolved within 48–72 hours as long as she maintained a gluten-free diet. Once she was well again, she would accurately recall the details of the prior images and again attempt to remain gluten-free. Since May 2012, she has had no further exposure to gluten and has remained symptom-free with no GI or CNS complaints.

Although her ATTG (antitissue transglutaminase) antibodies on a regular diet containing gluten were negative, she did not have testing for antigliadin antibodies, IgE antibodies directed against wheat proteins, anti-endomysial antibodies (EMA), or anddeamidatedgliadin, and she did not have an open or double-blind placebo-controlled challenge. An intestinal biopsy was also not performed. From an investigation perspective, the patient declined to have a complete workup for celiac disease, feeling that she was well as long as she maintained a gluten-free diet and that there was no further need for investigations.

## 3. Search Strategy and Selection Criteria

PubMed was searched with no date restrictions using the following terms to identify relevant literature: hallucinations, celiac, gluten sensitivity, and nonceliac gluten sensitivity. As expected in this emerging field of research, the majority of results were case reports, observational studies and clinical reviews.

## 4. Discussion

There have been multiple reports linking celiac disease and/or gluten sensitivity with mental health manifestations including isolated psychosis and full blown schizophrenia [7–10]. As in our case history, these cases report complete symptom resolution with removal of gluten. There is also evidence of frequent gluten sensitivity (but not celiac disease) in schizophrenic patients [4]. Furthermore, similar reports are published dealing with various other neurological manifestations in response to gluten exposure including “idiopathic” ataxia and neuropathies [11], epilepsy [12], mood swings [6], and autism [13]. In addition to neuropsychiatric phenomena, there are reports of other organ system involvement including reversible cardiomyopathy [14], resolved primary infertility [15], uveitis [16], and osteoporosis [17] in relation to the gluten exposure in celiac disease.

### 4.1. Diagnostic Criteria

In the recent literature, there has been a distinction drawn between those with CD and those with nonceliac gluten sensitivity (NCGS) [18–21]. The former denotes patients who typically have positive antibody titres and classic histological findings on intestinal biopsy, including crypt hyperplasia, villous atrophy, and an increase in intraepithelial lymphocytes [11]. NCGS, on the other hand, can be more difficult to diagnose. Although a group led by Gibson et al. in Australia first published a double-blind, randomized study confirming the reality of NCGS as a bona-fide medical condition [22], diagnostic criteria continue to be debated as the diagnosis is essentially determined as a result of self-reporting by patients. In general, these individuals have normal intestinal biopsies; they predictably react to gluten and have variable results with ATTG, anti-gliadin, and anti-EMA titres [2, 18, 19, 23]. Select private laboratories offer non-traditional stool testing to assess for antibodies and malabsorption secondary to gluten sensitivity; however, these tests have yet to be incorporated into formal diagnostic criteria and remain a controversial area requiring further investigation [24]. In fact, European studies to date studying stool antibody tests have demonstrated negative results [25].

NCGS patients demonstrate symptom improvement or resolution on a gluten-free diet and relapse with gluten challenge. Sapone et al. recently proposed an algorithm to differentiate between CD and NCGS. They suggest a diagnosis of NCGS can be made when symptoms are suggestive of CD, antibody titres are negative, and patients are symptomatic with gluten challenge, but this has yet to be widely accepted in the medical community and, unlike the description of other authors, requires all serology to be negative [23].

### 4.2. Acceptance by Health Care Professionals

There has been a tendency by some to attribute NCGS to placebo effect or somatization, particularly as the diagnosis is based on subjective self-reporting by patients. As well as the initial study confirming NCGS by Gibson et al. [22], however, an interesting study was recently published where groups of patients with CD, NCGS and a control group underwent complete psychiatric assessment and a subsequent gluten challenge [26]. There was found to be no difference between groups in their tendency to somatization, personality traits, or anxiety and depression symptoms. Moreover, patients with NCGS reported more symptoms than CD patients when challenged with gluten, suggesting NCGS to be a credible physical diagnosis [26].

As the evidence continues to accumulate, there does appear to be an increasing acceptance of this disease entity. A 2012 poll of nearly 1000 medical professionals reported that greater than 60% accepted the existence of NCGS and a follow-up article encouraged physicians to diagnose NCGS in patients who reacted to gluten and in whom celiac disease and wheat allergy had been excluded [27, 28]. A recent study, however, has suggested that NCGS may be more complex than previously realized. This study showed that after dietary reduction of fermentable, poorly absorbed, short-chain carbohydrates or FODMAPS (fermentable oligosaccharides, disaccharides, monosaccharides, and polyols), most participants were unable to detect gluten-specific effects [29]. The findings suggest that NCGS may not always be an isolated condition and may involve interaction, potentiation, or overlap with other types of sensitivities.

### 4.3. Causative Pathophysiological Mechanisms

**Figure 1:**
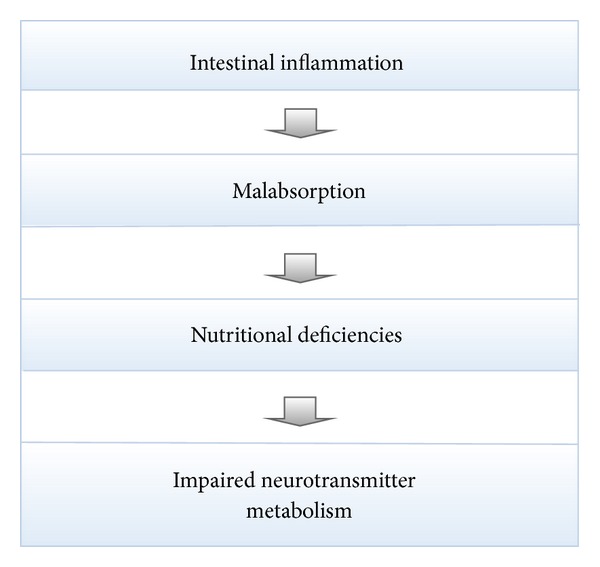
A potential pathophysiological mechanism associated with gluten sensitivity.

It is often thought that gluten induces systemic effects through inflammation of the intestinal tract. This is believed to cause malabsorption of various nutrients, leading to systemic deficiencies. For example, in the case of psychiatric illness, it is thought that there is perhaps impaired absorption of tryptophan, a precursor to serotonin, leading to serotonin deficiency and the presentation of mental illness [7] (see Figure 1). This explanation, however, is insufficient, given that not all patients reacting to gluten demonstrate intestinal changes and/or malabsorptive syndromes [3, 8, 26].

There have been numerous other theories proposed, which implicate specific antibodies, food allergies, and/or genetics in the pathophysiology of gluten-induced disease states [2, 30–35]. These mechanisms are primarily disease and/or system-specific and thus fail to provide an explanation of how gluten ingestion can lead to symptoms in multiple organ systems. Moreover, not all patients claiming to be sensitive to gluten have any family history or known genetic predisposition to celiac disease. To date, these theories remain primarily speculative and further research is required to delineate their plausibility.

**Figure 2:**
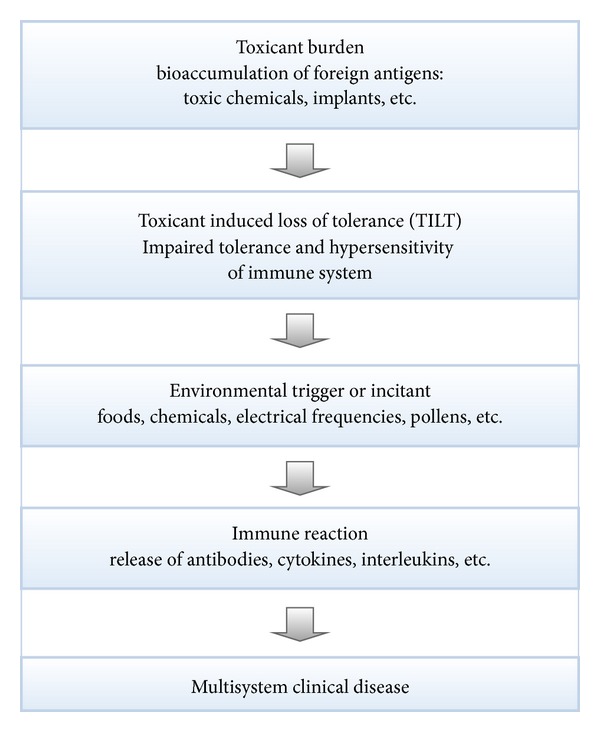
Sensitivity related illness: a causative pathway to multimorbidity.

An alternative, and perhaps more likely explanation, is that of sensitivity-related illness (SRI) (Figure 2) [36]. This mechanism of disease has recently been described and discussed in the scientific literature [36–42], whereby accumulated toxic insults often resulting from adverse chemical exposures lead to hypersensitivity and impaired tolerance of the immune system (known as toxicant induced loss of tolerance or “TILT”). With growing attention in the medical literature to the escalating problem of toxicant exposure and bioaccumulation within contemporary society [43–45], this mechanism of illness has become compelling indeed. Notable groups such as the World Health Organization and the Centers for Disease Control have recently drawn attention to the reality of ubiquitous toxicant exposures and the chemical erosion of human health associated with toxicant accrual within the human body [43–45].

After the bioaccumulation of a toxicant burden and the consequent immune dysregulation [36], seemingly insignificant environmental triggers can lead to the release of proinflammatory cytokines [46], antibodies, chemokines, and interleukins and produce a variety of symptoms, including neuropsychiatric issues, in the affected patient [36, 40, 47, 48]. Gluten is one such common trigger, and is hypothesized to be the culprit in the above case report. With the ability of SRI to induce multisystem manifestations and with its increasing and widespread prevalence [49–52], this mechanism of disease is the preferred explanation of the authors for gluten-induced neuropsychiatric disease. As patients with SRI typically have multiple triggering agents, most commonly including gluten, casein, and sugar, this causative explanation would also address the uncertainty about NCGS that was highlighted in an editorial by Vanga and Leffler in *Gastroenterology* [53]. This mechanism also explains the apparently inexplicable onset of gluten sensitivity in patients who were previously well and fully tolerant of gluten and accounts for the reversal of gluten sensitivity in some patients who are successful in eliminating their toxicant burden [36].

## 5. Concluding Thoughts

There are many theories and hypotheses as to the origins of recurrent hallucinations including genetic illness, metaphysical attribution, and neurochemical disruption. The profound impact of such neuropsychiatric dysregulation on the lives of those afflicted is evident. Concomitant with this reality is escalating evidence about sensitivity-related disorders including intolerance and multimorbidity associated with gluten exposure. The widespread prevalence of gluten in the typical North American diet and the escalating numbers of published case reports linking gluten exposure with a myriad of manifestations raise awareness of a potential modifiable lifestyle determinant that may have a substantial impact on some individuals with neuropsychiatric and other health problems.

The individual in the presented case demonstrates a clear sensitivity to gluten with remission of longstanding hallucinations with gluten elimination and relapsing symptoms upon reintroduction of dietary gluten. The scientific literature contains numerous case reports where unexplained symptoms are significantly improved and, at times, completely resolved when similar dietary changes are made. Therefore, when clinicians are faced with physical symptoms that have not been otherwise explained, celiac testing may be warranted. If this is found to be negative, the possibility of NCGS and SRI ought to be considered. Although NCGS cannot be definitively diagnosed at this time based on laboratory investigations, a trial of gluten elimination should be incorporated as part of the clinical assessment and potential management.

## Key Points

1. Gluten ingestion in gluten sensitive individuals can lead to a variety of clinical presentations including psychiatric, neurological, gynecological, and cardiac symptoms.
2. Dietary elimination of gluten may lead to complete symptom resolution.
3. Health practitioners are advised to consider gluten elimination in patients with otherwise unexplained symptoms.
4. Nonceliac gluten sensitivity may be a part of a constellation of symptoms resulting from a toxicant induced loss of tolerance (TILT).
